# Phytoplankton Assemblage Characteristics in Recurrently Fluctuating Environments

**DOI:** 10.1371/journal.pone.0120673

**Published:** 2015-03-23

**Authors:** Daniel L. Roelke, Sofie Spatharis

**Affiliations:** 1 Texas A&M University, Department of Wildlife and Fisheries Sciences, and Department of Oceanography, 2258 TAMUS, College Station, Texas 77843–2258, United States of America; 2 University of Glasgow, Institute of Biodiversity, Animal Health and Comparative Medicine, Glasgow, G12 8QQ, Scotland, United Kingdom; University of Sydney, AUSTRALIA

## Abstract

Annual variations in biogeochemical and physical processes can lead to nutrient variability and seasonal patterns in phytoplankton productivity and assemblage structure. In many coastal systems river inflow and water exchange with the ocean varies seasonally, and alternating periods can arise where the nutrient most limiting to phytoplankton growth switches. Transitions between these alternating periods can be sudden or gradual and this depends on human activities, such as reservoir construction and interbasin water transfers. How such activities might influence phytoplankton assemblages is largely unknown. Here, we employed a multispecies, multi-nutrient model to explore how nutrient loading switching mode might affect characteristics of phytoplankton assemblages. The model is based on the Monod-relationship, predicting an instantaneous growth rate from ambient inorganic nutrient concentrations whereas the limiting nutrient at any given time was determined by Liebig’s Law of the Minimum. Our simulated phytoplankton assemblages self-organized from species rich pools over a 15-year period, and only the surviving species were considered as assemblage members. Using the model, we explored the interactive effects of complementarity level in trait trade-offs within phytoplankton assemblages and the amount of noise in the resource supply concentrations. We found that the effect of shift from a sudden resource supply transition to a gradual one, as observed in systems impacted by watershed development, was dependent on the level of complementarity. In the extremes, phytoplankton species richness and relative overyielding increased when complementarity was lowest, and phytoplankton biomass increased greatly when complementarity was highest. For low-complementarity simulations, the persistence of poorer-performing phytoplankton species of intermediate R*s led to higher richness and relative overyielding. For high-complementarity simulations, the formation of phytoplankton species clusters and niche compression enabled higher biomass accumulation. Our findings suggest that an understanding of factors influencing the emergence of life history traits important to complementarity is necessary to predict the impact of watershed development on phytoplankton productivity and assemblage structure.

## Introduction

Coastal lagoons, estuaries and bays represent some of the most productive ecosystems on the planet and are comprised of habitats important to many of the world’s species [1]. The high productivity and biodiversity found in these coastal systems are influenced, in part, by phytoplankton productivity and assemblage composition. In turn, phytoplankton are influenced by a complex interplay among many factors that sometimes include inorganic nutrients, light, temperature, stratification, grazing and allelopathy [2–4]. Thus, factors acting at the base of the food web, such as these, can ultimately influence ecosystem form and functioning.

Regarding inorganic nutrients, annual variations in freshwater inflow to coastal systems are known to influence their availability. Similarly, annual variations in water exchange with the ocean are known to influence the availability of nutrients. The net effect of annually varying freshwater inflows and ocean water exchanges can lead to alternating periods where the nutrient most limiting to reproductive growth switches [5–10]. Shifts in nutrient availabilities, like this, can influence productivity and composition of phytoplankton. This phenomenon has been observed regularly in inland water bodies, and an understanding of the underpinning mechanisms has been well developed through empirical and theoretical research [11–14].

In coastal systems where it occurs, the mode of switching between which nutrient is most limiting to reproductive growth likely differs. For example, in systems characteristic of abrupt changes in nutrient loadings, sudden transitions in which nutrient is the most limiting might arise. Abrupt changes in nutrient loadings might follow short-period runoff events associated with precipitation [15–17] or short-period vertical mixing events associated with winds [18–20]. On the other hand, in systems that are characteristic of seasonally developing wet and dry periods or annually occurring periods of upwelling [21, 22], gradual changes in nutrient availabilities might arise.

Human activities might also influence the mode of switching between limiting nutrients. For example, coastal systems receiving inflows from watersheds with many reservoirs might experience gradual changes in nutrient availabilities. This would occur because during reservoir operations peak in-stream flows tend to be lower relative to historical (pre-dam) conditions and low in-stream flows tend to be elevated, resulting in a less dynamic flow regime [23, 24]. Another human activity that might influence the mode of switching between limiting nutrients is interbasin water transfers to basins with water shortages from basins with water surpluses. During interbasin water transfers flow through the watershed receiving the water can be elevated, while flow through the watershed donating the water can be reduced, with both scenarios resulting in a less dynamic condition.

While phytoplankton productivity is linked to nutrient loading associated with inflows and ocean water exchanges, it is better characterized by cellular nutrient uptake and conversion to biomass, which is the process of resource exploitation. As an ecological principle, the level of complementarity in an assemblage influences the degree of resource exploitation. Here, complementarity refers to greater resource exploitation facilitated by the interaction between co-occurring species [25–27]. Sometimes the level of complementarity can lead to overyielding. This is a condition where the productivity of the assemblage is greater than the average productivity of all members of the assemblage when they are each considered in monoculture [28, 29]. In the extreme, overyielding can be transgressive. This is a special condition where the productivity of the assemblage is greater than the productivity of the most productive member of the assemblage when it is considered in monoculture [30].

In this research, we employ a multispecies, multi-nutrient mathematical model to explore how nutrient loading switching mode, i.e., sudden vs. gradual, might effect notable phytoplankton assemblage characteristics. These included time-averaged richness, evenness, total biomass, degree of overyielding, species interactions, length of the resource trade-off space exploited, and resource utilization. These characterizations are focused upon because they are often used to describe ecosystem form and functioning. In addition, we investigated the influence of the level of complementarity and the amount of noise in the resource supply concentration on these assemblage characteristics. Such information will increase our understanding of how phytoplankton systems might respond to increased landscape alterations driven by human development and population growth. To keep our model tractable, we focus solely on competition for nutrients limiting to reproductive growth and its relationship with assemblage characteristics. Other notable factors, such as temperature, stratification, light, grazing and allelopathy, are not studied here. This is not to say that these other factors are unimportant to the phytoplankton assemblage characteristics studied here. Indeed, the compounding affects of these other factors should be a focus of future studies.

## Methods

Our multispecies, multi-nutrient model was governed by the widely-used Monod-relationship, which predicts an instantaneous reproductive growth rate from ambient inorganic nutrient concentrations [31]. This relationship is defined by two life history traits. The first is the maximum specific reproductive growth rate (*μ*_*max*_). The second is the half-saturation coefficient for reproductive growth (*k*_*S*_). Together, these life history traits enable construction of a curve that asymptotically approaches a species-specific maximum reproductive growth rate as nutrient concentrations increase. The rate at which this curve approaches the maximum reproductive growth rate is determined by the initial slope of this curve, which is defined by 0.5*μ_max_ divided by k_S_. Because our model employed two reproductive growth-limiting nutrients, we used Liebig’s Law of the Minimum to determine which inorganic nutrient was limiting to each species at any point during model simulations [31]. As we will describe in the sections below, we combined these relationships with a population loss factor, i.e., hydraulic displacement of cells associated with inflows, to characterize each species’ niche using the R* conceptual model [11], thus enabling an ecological interpretation of our modeling results.

The phytoplankton assemblages employed in our analyses were the result of self-organization processes where surviving species emerged from initial species-rich pools [32]. The initial species pools varied in their level of complementarity among species (discussed further below). Which species survived the self-organization process was determined, in part, by their competitive abilities for the two reproductive growth-limiting nutrients, which is reflected by their R* values (lower R* values indicate better competitive abilities). Here, the half-saturation coefficients for resource-limited reproductive growth (*k*_*S*_) and the fixed cellular content of resources (*Q*_*S*_) were the life history traits that differentiated competing species. During self-organization, two modes of resource supply fluctuation were explored, where resource supply transitions were either sudden or gradual (discussed further below). Once self-organized phytoplankton assemblages emerged under these fluctuation modes, we compared characteristics of the assemblages that included time-averaged richness, evenness, total biomass, degree of overyielding, species interactions, length of the resource trade-off space exploited, and resource utilization.

### Mathematical model and numerical solution

We employed a well-known mathematical model developed by Tilman [11] for depicting population dynamics of primary producers, i.e., plants, macroalgae and phytoplankton [11, 13, 32]. We structured the model to simulate a phytoplankton assemblage where new resources arrived with inflow, and loss of cells and ambient nutrients occurred through hydraulic flushing.

For each species, population dynamics were simulated using an equation of the form:

$$\frac{dN}{dt}=\mu N-vN$$ (1)

where *N* was population density (cells liter^-1^), *μ* was specific reproductive growth rate (d^-1^), and ν was hydraulic flushing defined as the inflow divided by the system volume (d^-1^). For the purposes of this research, we assume that carbon content per cell is constant, so *N* becomes an analog of biomass.

As mentioned above, specific reproductive growth rate for each population was determined using the Monod equation and Liebig's "Law of the Minimum" [33] following the form:

$$\mu =\mu_{max}(min[\frac{S1}{S1+k_{S1}},\frac{S2}{S2+k_{S2}}])$$ (2)

where *μ*_*max*_ was the maximum specific reproductive growth rate for a species (d^-1^), *S1* and *S2* were the concentration of resources necessary for reproductive growth (*μ*M), and *k*_*S1*_ and *k*_*S2*_ were the half-saturation coefficients for resource-limited reproductive growth (*μ*M) specific to each species. A function ‘min’ was used to determine which resource was more strongly limiting to reproductive growth at each time step of the simulation.

For the two resources necessary for reproductive growth, changes in concentration were simulated using an equation of the form:

$$\frac{dS}{dt}=v(S_{source}-S)-\sum_{i=1}^{n}Q_{s_{i}}\mu_{i}N_{i}$$ (3)

where *S*_*source*_ was the fixed concentration of the resource in the source (*μ*M), $Q_{s_{i}}$ was the fixed cellular content of the resource (*μ*mole cell^-1^) for a species, *n* was the total number of species, and other parameters were the same as previously described.

Differential equations were solved numerically using ordinary differential equation solving routines that were a part of a commercial software package (The Math Works, Inc.). The routines were based on fourth-order Runge-Kutta procedures, and used a variable time step that was based on a local error tolerance set at 10^-15^.

### Fluctuations in the resource supply

Assemblages self-organized under an annually fluctuating resource supply (*S*_*source*_). Simulations were initiated with the first resource having a concentration of 2 *μ*M and the second resource a concentration of 20 *μ*M. These are within the range of resource concentrations observed for river discharges into coastal lagoons, estuaries and bays where anthropogenic eutrophication is not as prevalent [34]. Over a period of ~182 days, the concentrations of the resources in the supply reversed. That is, at day 182 the first resource had a concentration of 20 *μ*M and the second resource a concentration of 2 *μ*M.

**Fig 1:**
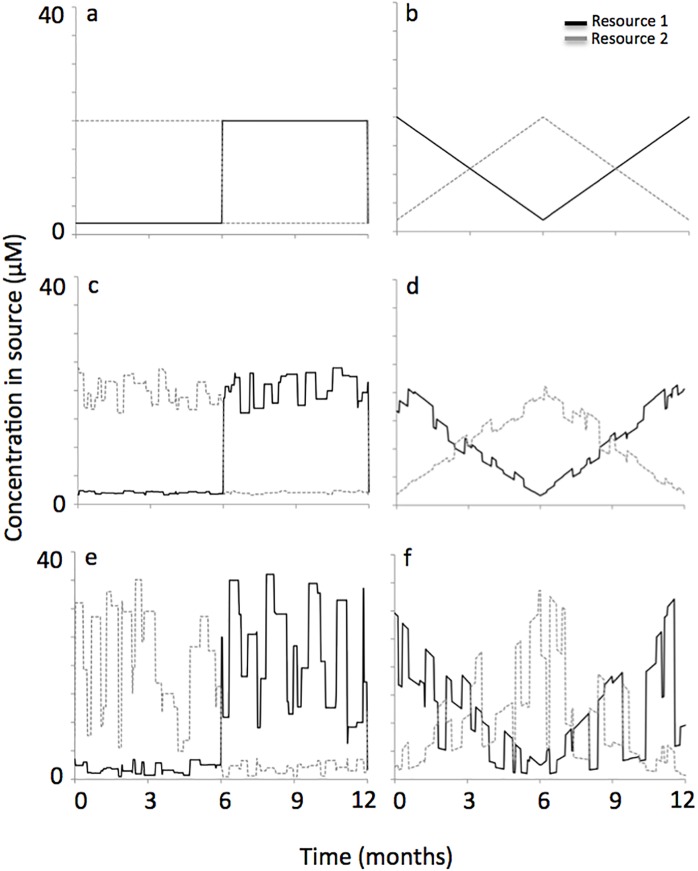
Sudden and gradual modes of reversal in the resource supply concentration. The sudden mode of reversal went from a resource supply of 2 and 20 *μ*M for resources 1 and 2 to a resource supply of 20 and 2 *μ*M with an abrupt transition on day 182. Three levels of noise in the resource supply were explore, which were no noise (a), 0–20% noise (c) and 0–80% noise (e). The gradual mode of reversal also went from a resource supply of 2 and 20 *μ*M for resources 1 and 2 to a resource supply of 20 and 2 *μ*M, but with a slow change that reversed direction on day 182. Again, three levels of noise in the resource supply were explore, which were no noise (b), 0–20% noise (d) and 0–80% noise (f).

We explored two modes of this resource supply reversal. The first mode was a sudden change, where resource supply concentrations were 2 and 20 *μ*M for days 1 to 182, then abruptly switched to 20 and 2 *μ*M on day 182 and remained at those concentrations through day 365. On day 365, they abruptly switched back to concentrations of 2 and 20 *μ*M (see Fig. 1a). The second mode of resource supply reversal was a gradual change, where the first resource progressively increased from 2 *μ*M on day 1 to 20 *μ*M on day 182, while the concentration of the second resource progressively decreased from 20 *μ*M on day 1 to 2 *μ*M on day 182. From days 182 through 365 the resources progressively changed back to their initial values (see Fig. 1b). Thus, the fluctuation period for both modes of resource supply reversal occurred over an interval of 365 days. As mentioned previously, switching between limiting resources over a period of a year is observed in many natural systems, especially when the year is characterized by wet and dry seasons [5–10].

In natural settings, however, it is unlikely that resource supply reversals are quite so regular. Consequently, we performed additional simulations with added noise to the resource supply. We created a function that randomly altered the resource concentration in the supply by adding or subtracting a value equivalent to some percentage of the resource supply concentration that was used for the ‘regular’ scenarios (described in the preceding paragraph and shown in Fig. 1a, b). The function also accounted for the duration that the resource supply was altered. More specifically, the adjusted resource concentration in the supply was randomly varied in magnitude over the interval (-20%, 20%) (see Fig. 1c, d for representative simulations) or over the interval (-80%, 80%) (see Fig. 1e, f for representative simulations). This resource supply adjustment, or amount of noise in the resource supply concentration, occurred over periods that randomly varied in duration from 1 to 14 days. The noise pattern did not repeat with each annual cycle. In other words, each simulation year of each 15-year period simulated was unique. The noise pattern was also unique between each of the 100 replicate simulations per scenario (discussed below). We selected these intervals for noise because they generated simulation results for resource supply variability similar to what is observed in multiple coastal systems [15, 35–37].

### Parameterization of populations to produce self-organized, species-rich assemblages

**Fig 2:**
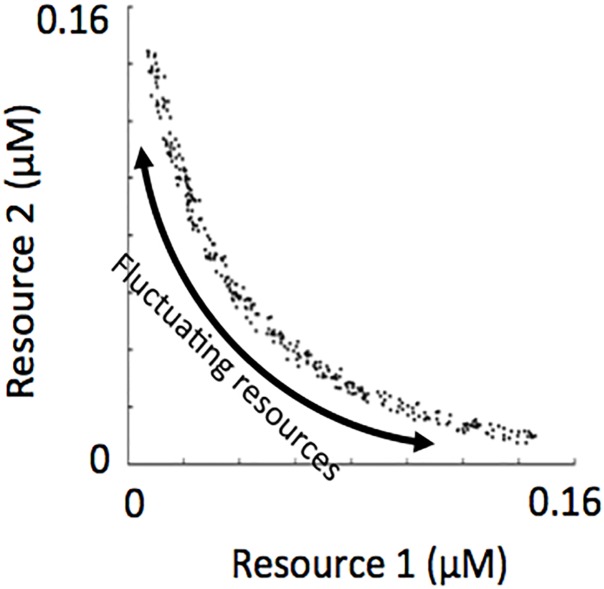
R*s of phytoplankton in the initial species pool and the recurrently fluctuating environmental conditions from which assemblages self-organized.

We generated phytoplankton assemblages using a numerical approach that involved self-organization from a species-rich pool under fluctuating resource supply conditions, where the number of species in the initial pool was 300 and they were distributed throughout the resource trade-off space (Fig. 2). The authors have worked in a variety of aquatic ecosystem types that include lakes, rivers, bays and coastal oceans [35, 38–40]. Field sampling in these systems usually spanned multiple seasons, with sampling frequencies ranging from weekly to quarterly. Phytoplankton species richness in these ecosystems ranged from one to ~80, with 15–20 species being the norm. Thus, we considered the initial species pool of 300 used in these simulations as “species rich”. The simulated period of self-organization was 15 years. Surviving species after this simulated period (those with biomass >0.05% of the total biomass, a level chosen because oscillating populations at this point in the model did not decrease to levels below this [41] were then used to initiate a second 15-year simulation. The surviving species from this second simulation were considered members of a self-organized assemblage. In this way, we assured ourselves that the processes underlying coexistence in our simulations followed from life history traits and not potential effects from an initial species-rich pool.

Parameterization of life history traits in the species-rich pool, i.e., reproductive growth-related factors in our simulations, were not random. We used our knowledge of physiological traits and basic ecological principles as a guide. For example, competitive ability for a specific resource was inversely proportional to a species’ cellular resource content [42]. In other words, the resource-specific, half-saturation coefficient for reproductive growth (*k*_*S*_) and the cellular resource content (*Q*_*S*_) varied proportionally. To illustrate, *k*_*S*_ was selected randomly from numbers within the range 0.04–1. When in units of *μ*M this range represents what is typically observed in phytoplankton for half-saturation coefficients [14, 43]. Following the principle of proportionality mentioned above, *Q*_*S*_ was then set equal to *k*_*S*_. When in units of 10^-6^ *μ*mole cell^-1^ the range of 0.04–1 represents typical resource contents measured in phytoplankton [14, 43]. Therefore, species with relatively low *k*_*S*_ and *Q*_*S*_ in our simulations represented populations with high competitive ability and low demand for that resource. Conversely, species with relatively high *k*_*S*_ and *Q*_*S*_ represented populations with low competitive ability and high demand for that resource.

Our parameterization of reproductive growth-related factors was further guided by a trade-off between competitive abilities for the two resources used in the model. A species being a good competitor for one resource meant that it was a poor competitor for the other resource; and a species being an intermediate competitor for one resource meant that it was also an intermediate competitor for the other resource [11, 44]. This prevented any single species from being a superior competitor for both resources. To illustrate, a species with *k*_*S1*_ and *Q*_*S1*_ values of 0.15 for resource 1 would then have *k*_*S2*_ and *Q*_*S2*_ values of 0.85 for resource 2. This species would be characterized as a good competitor for resource 1 and a poor competitor for resource 2. A species with *k*_*S1*_ and *Q*_*S1*_ values of 0.45 for resource 1 would then have *k*_*S2*_ and *Q*_*S2*_ values of 0.55 for resource 2. This species would be characterized as an intermediate competitor for resources 1 and 2.

### Complementarity level in the initial species pool and the R* model framework

Initial species pools were generated with varied levels of complementarity (twelve total). Here, complementarity refers to time-averaged resource use by a self-organized assemblage under conditions of resource supply fluctuation over an annual cycle. Assemblages that are able to use more resources, i.e., draw down ambient resource concentrations to lower levels, are considered more complementary.

To provide a mechanistic understanding of how complementarity in the assemblage affected other characteristics of the self-organized assemblage (discussed further below), species were analyzed based on one of their life-history traits, *k*_*S*_ (recall that the other life history trait that varied among species was *Q*_*S*_, which was proportional to *k*_*S*_). Along with *μ*_*max*_ and *v*, which were the same for all species in the assemblage, knowledge of *k*_*S*_ enabled determination of R* values for each species [11, 12, 45]. This was achieved following the equation:

$$R*=\frac{\nu k_{s}}{\mu_{max}-\nu }$$ (4)

Our simulations employed two resources therefore the resource trade-off space was two-dimensional.

**Fig 3:**
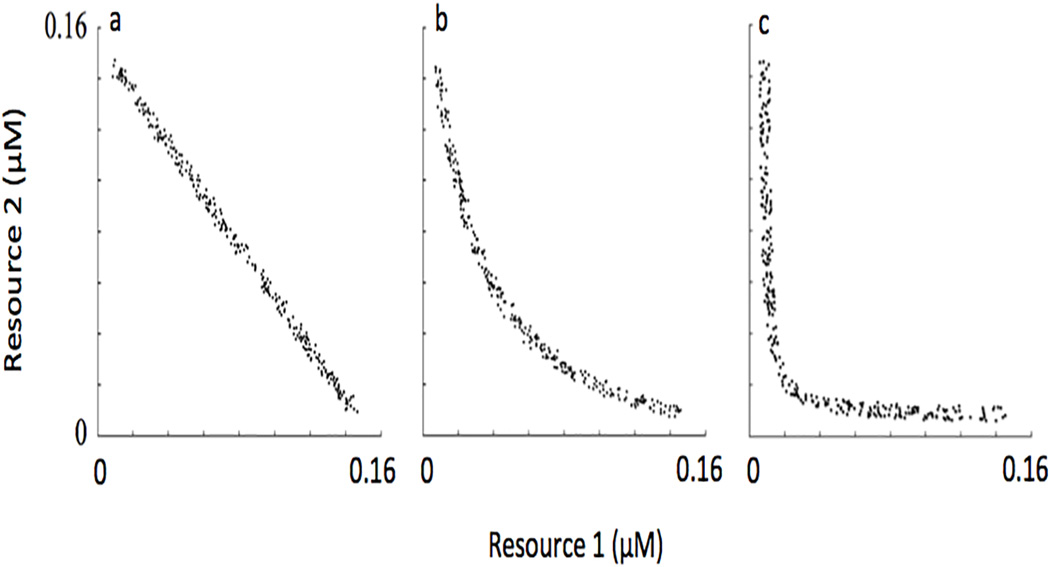
Representative R* species distributions in the initial pool of 300 populations at the lowest (a), intermediate (b) and highest (c) level of complementarity.

As mentioned previously, we employed a proportional resource trade-off when assigning species specific values of *k*_*S*_ for the two resources. Consequently, the distribution of species’ R*s through a two-dimensional resource trade-off space was linear (see Fig. 3a). Linear distributions of R*s were not commonly observed in plankton systems, rather distributions of R*s with a downward curve were more commonly observed [42]. To generate assemblages with a downward-curved distribution of R*s (see representative assemblages in Fig. 3b, c), we transformed the R*s from a linear distribution into a downward-curved distribution following a simple procedure described in the next section.

Conceptually, assemblages with linear distributions of R*s through the resource tradeoff space should use less resources over time compared to assemblages with downward-curved distributions of R*s through the resource tradeoff space. As mentioned above, we defined complementarity as the time-averaged resource use by a self-organized assemblage under conditions of resource supply fluctuation over an annual cycle. Consequently, we now refer to assemblages with linear distributions of R*s as having the lowest level of complementarity and assemblages with the greatest downward-curved distributions of R*s as having the highest level of complementarity.

### Shape function and levels of complementarity

The shape function used for manipulating the level of complementarity within an assemblage followed the form:

$$y=\frac{a}{x}$$ (5)

where *a* was a user defined coefficient that controlled the shape of the curve for the region defined over the intervals *x*[0,1] and *y*[0,1]. To illustrate, when *a* = 0.5 a range of *y* values from 1 to 0.5 are possible in the interval x[0,1]. The curve of the line in this region dips towards the origin (for example, compare Fig. 3b where the line is “dipped” with Fig. 3a where the line is linear). As *a* decreases, the lower range of possible *y* values also lowers, and the grade to which the curve dips towards the origin becomes more pronounced (for example, compare Fig. 3c with Fig. 3b). Using a polynomial fitting function that was a part of a commercial software package (The Math Works, Inc.), the life-history traits of the populations in the initial species pool were transformed so that their distribution through the trade-off space matched the curve of the shape function.

In this research, 12 levels of assemblage complementarity were explored. These corresponded to *a* values of 0.99 (lowest complementarity level), 0.82, 0.66, 0.50, 0.39, 0.28, 0.17, 0.13, 0.09, 0.05, 0.03 and 0.01 (highest complementarity level). These values for *a* resulted in regular spaced resource trade-off curves through the resource trade-off space. For each complementarity level, 100 self-organized assemblages were generated. Here, the number of replications was determined by calculating the average and standard deviation of our assemblage characteristics of interest (see further below) with each additional simulation. We stopped replication at 100 because the means and standard deviations for the assemblage characteristics of interest no longer varied with additional simulations. In total, 1200 simulations were performed.

Prior to the simulations mentioned above, a low amount of noise was added to each life history trait not exceeding 0.04. This is why the species’ R*s do not line up exactly along the resource trade-off curves shown in Fig. 3. In a natural system, it is not likely that R*s would line up exactly on a line through the resource trade-off space. Adding some noise to *k*_*S*_ and *Q*_*S*_ added realism to the model. In this research, we did not explore the relationship between the amount of noise added to the life history traits and the assemblage characteristics of interest.

### Model initialization

Initial population densities for each species were the same for all simulations, i.e., *N* = 0.1 (x10^6^ cells liter^-1^). Initial resource concentrations were 2 *μ*M for resource 1 and 20 *μ*M for resource 2. Parameter constants included total flushing and maximum specific reproductive growth rate, which were *v* = 0.25 d^-1^ and *μ*_*max*_ = 2 d^-1^. As mentioned previously, values for *k*_*S*_ were within the range 0.04 and 1.0 (*μ*M), and values for *Q*_*S*_ ranged within 0.04 and 1.0 (10^-6^ *μ*mole cell^-1^). All parameterizations were within the range of what is typical for phytoplankton assemblages and pelagic environments [14, 43].

### Assemblage characteristics of interest

For purposes of this research, eight characteristics were determined for each self-organized phytoplankton assemblage. These included time-averaged richness (number of coexisting species), evenness (based on biomasses of co-occurring populations), total biomass (a sum of co-existing populations’ cell density, in units of x10^6^ cells liter^-1^), degree of overyielding, species interactions, length of the resource trade-off space exploited, and resource utilization. Here, ‘time-averaged’ means that the characteristic of interest was based on either the population densities or ambient resource concentrations averaged over the last year of the 15-year period. The simulated period on which to focus our analyses (15^th^ year) was determined by observing a recurrent annual cycle in population dynamics [41], thus minimizing potential effects of transient dynamics common during the initial years of the simulations.

Total biomass (*O*_*T*_) and species richness (*S*) were the total number of cells present and the number of coexisting species respectively. An evenness index (*J*) was determined following Pielou [46], which was:

$$J=\frac{H'}{ln(S)}$$ (6)

$$H'=-\sum_{i=1}^{S}p_{i\;}ln(p_{i})$$ (7)

where *H’* was a diversity index and *p*_*i*_ was the probability that any given cell was species *i* [47], and other parameters were the same as previously defined.

The maximum deviation yield (*D*_*max*_), an indicator of transgressive overyielding when >0 (i.e. the yield of the mixture exceeds that of the most productive monoculture), was determined following:

$$D_{max}=\frac{O_{T}-max(M_{i})}{max(M_{i})}$$ (8)

where *O*_*T*_ was the same as previously defined and max(*Mi*) was the population density of species *i* observed in the most productive monoculture under the same conditions as the self-organized assemblage [30].

Not all species in an over-yielded assemblage contributed to the overyielding. To determine which species contributed to overyielding in our self-organized assemblages, and the degree to which they contributed, the species-specific yield exponent (*y*_*i*_) was calculated using:

$$y_{i}={log}_{s}(\frac{O_{i}}{M_{i}})$$ (9)

where *O*_*i*_ was the population density for species *i* observed in the self-organized assemblage and *M*_*i*_ was the population density for species *i* observed in a monoculture under the same conditions as the self-organized assemblage [48, 49].

The deviation yield total (*D*_*T*_) is a measure of the proportional deviation of the observed total yield from its expected value. This is a standardized way to compare the overall performance of a mixture with its expectation in the absence of species interactions. Thus larger values indicate greater species interactions. This metric was determined following:

$$D_{T}=\frac{O_{T}-E_{T}}{E_{T}}$$ (10)

$$E_{T}=\sum_{i=1}^{S}E_{i}$$ (11)

$$E_{i}=\tilde{p}M_{i}$$ (12)

$$\tilde{p}=\frac{1}{S}$$ (13)

where *O*_*T*_ was the same as previously defined, *E*_*T*_ was the sum of the expected probabilities from monocultures, *E*_*i*_ was the expected probability for each species based on monoculture, $\tilde{p}$ was the expected probability that any given cell belongs to a population assuming all populations were evenly represented in the total number of coexisting populations, and *M*_*i*_ was the same as previously defined [30].

The length of the resource trade-off space exploited was defined simply as the distance between the two species at either extreme of the resource trade-off curve, which we will now call R*distance. This was calculated using the Pythagorean Theorem. Here, R*distance was simply the hypotenuse of the triangle formed when the distance between the R*s for resource 1 of the two extreme species and the distance between the R*s for resource 2 of the two extreme species formed the legs of the triangle.

Finally, resource utilization was determined by averaging the concentration of a resource over the last year of the simulation, where lower averaged resource concentration reflected greater use of that resource. We calculated this for resource 1 and 2.

### Statistics

We employed 3-way ANOVAs to partition the variance of each of the assemblage characteristics (time-averaged richness, evenness, total biomass, degree of overyielding and species interactions) between the explanatory variables (resource supply reversal mode, noise level, complementarity level, and their interactions). This analysis was not used for hypothesis testing as p-values are bound to be minuscule due to increased sample sizes. Instead the F-ratios were used as a quantitative measure of the amount of variability explained by each explanatory variable (Table 1). Guided by ANOVA results, we performed two principal component analyses, the first using simulation results from the sudden reversal scenarios and the second using simulation results from the gradual reversal scenarios.

**Table 1:** F-ratios resulting from Analysis of Variance on the effects of fluctuation type, noise and complementarity levels and their interactions on multiple assemblage characteristics (e.g. biomass, richness, etc).

			*F-Ratio*							
		*Df*	*Species richness*	*Evenness*	*Biomass*	*Dmax*	*D*_*T*_	R* distance	*R1 min*	*R2 min*
Main effects	A:Reversal mode	1	5094	809	76284	1774	1762	1004	831	628
	B:Noise	2	1202	326	260	482	703	227	5262	13216
	C:Complementarity	11	161	11	14366	2718	364	1207	21	11
Interactions	AB	2	8	154	24	30	439	136	404	252
	AC	11	223	115	5256	241	349	69	6	8
	BC	22	88	22	21	8	66	14	6	13
	ABC	22	13	81	7	32	95	20	11	14

Reversal mode refers to the sudden and gradual mode of reversal of the two resources, noise refers to the three noise levels employed (zero, 0–20%, and 0–80%), and complementarity refers to the 12 complementarity levels.

## Results

**Fig 4:**
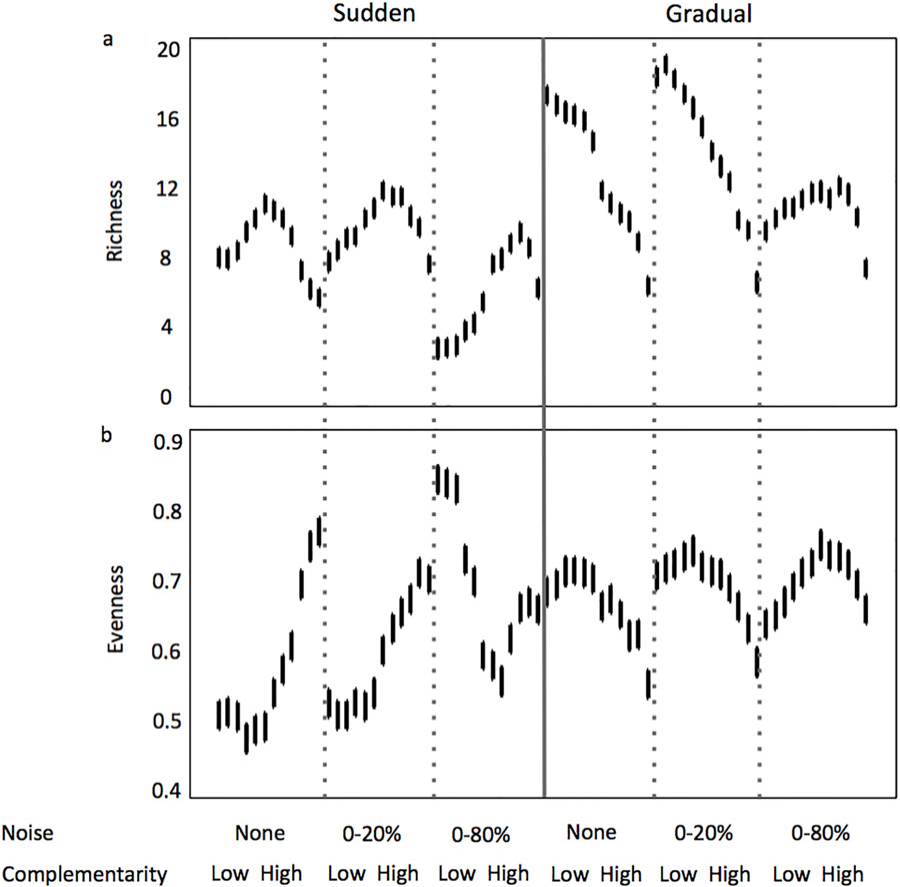
Variability of species richness (number of coexisting species) (a) and evenness (index based on biomasses of co-occurring populations) (b) along a gradient of increasing complementarity in the assemblages and noise in resource concentration from the supply, when the reversal in resource supply concentrations is sudden and gradual. Error bars represent 95% confidence intervals.

In almost all simulations, species-rich assemblages emerged from the process of self-organization (Fig. 4a). Here, species-rich refers to a condition where the number of coexisting populations was greater than the number of limiting resources. This occurred regardless of the mode of reversal in the resource supply concentrations, and in general, regardless of the amount of noise applied to the resource supply concentrations. Exceptions occurred in the sudden reversal scenarios when complementarity level was low and the amount of noise in the resource supply was 0–80%.

The mode of reversal in the resource supply concentration explained the most variation in time-averaged richness and evenness. This was followed by the amount of noise in the resource supply concentrations, with the level of complementarity explaining the least amount of variability in these assemblage characteristics (Table 1).

For time-averaged richness, when the mode of reversal in the resource supply concentrations was sudden, the highest time-averaged richness was ~12. When the mode of reversal was gradual, the highest time-averaged richness was ~19 (Fig. 4a). Adding noise to the resource supply concentrations at the 0–20% amount had little effect. But adding noise at the 0–80% amount reduced the time-averaged richness by as much as 50%. The greatest differences between sudden and gradual reversal modes in resource supply concentrations occurred when complementarity level was low, where time-averaged richness was 2- to 3-fold greater in the gradual resource supply reversal.

**Fig 5:**
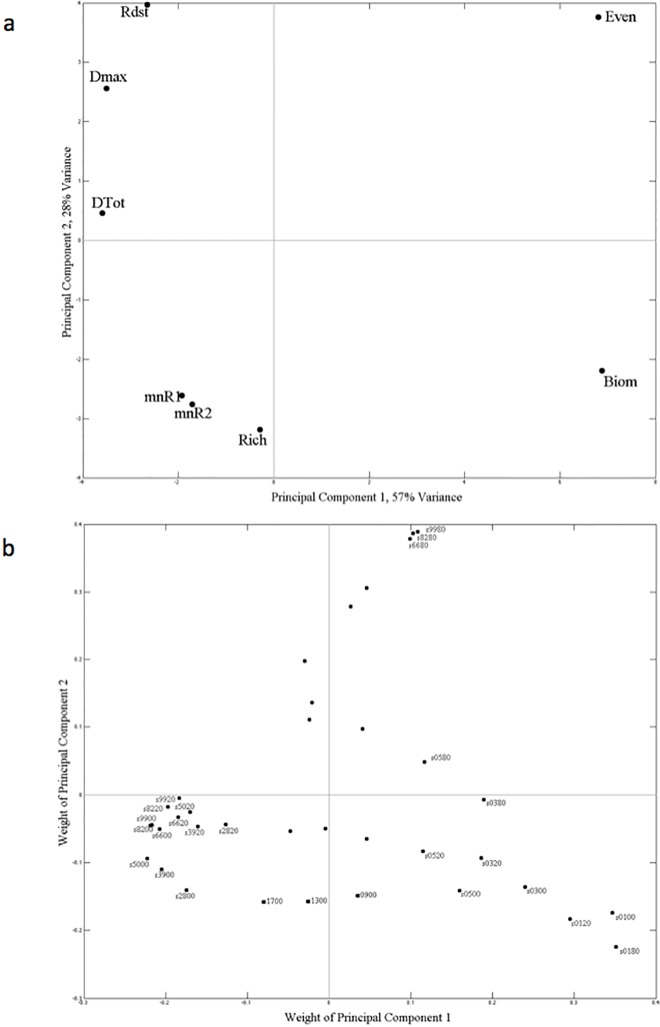
Principal component analysis using all simulations where the transitions in resource supply concentration were sudden. Parameter weightings (a) and the simulation scenario weightings (b) on the two principal components with the greatest eigenvalues, PC1 and PC2, represented 57% and 28%, respectively, of the total variability. Parameters in the analysis were timer-averaged richness (‘Rich’), evenness (‘Even’), biomass ‘(Biom’), overyielding (‘Dmax’), species interactions (‘DTot’), length of the resource trade-off curve exploited (‘Rdst’), and drawdown of resources one and two (‘mnR1’ and ‘mnR2’). Simulation scenarios are identified using a numbering scheme where the first two digits identify the level of complementarity (‘99’ lowest level, ‘01’ highest level) and the second two digits identify the level of noise in the resource supply concentrations (‘00’ means no noise, ‘20’ means 0 to 20% noise, and ‘80’ means 0 to 80% noise).

The relationship between complementarity level and time-averaged richness also varied between sudden and gradual reversal modes in the resource supply concentrations (Fig. 4a). For scenarios where the mode of reversal was sudden, the relationship was unimodal. In those scenarios, the complementarity level where time-averaged richness maxima occurred shifted towards higher levels of complementarity as the amount of noise increased. This shift came about because of the large decreases in richness that occurred at lower complementarity levels with the 0–80% added noise. This latter trend weighted heavily on principal component 2 (~28% of total variability) in the PCA analysis focused on sudden reversal scenarios (Fig. 5).

**Fig 6:**
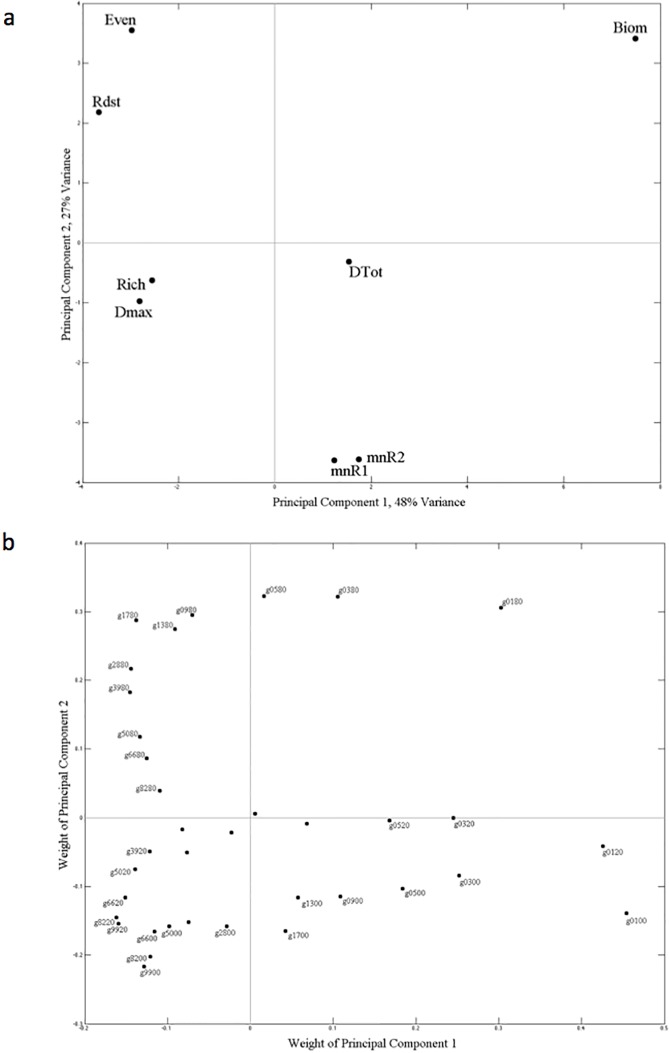
Principal component analysis using all simulations where the transitions in resource supply concentration were gradual. Parameter weightings (a) and the simulation scenario weightings (b) on the two principal components with the greatest eigenvalues, PC1 and PC2, represented 48% and 27%, respectively, of the total variability. Parameters in the analysis were timer-averaged richness (‘Rich’), evenness (‘Even’), biomass ‘(Biom’), overyielding (‘Dmax’), species interactions (‘DTot’), length of the resource trade-off curve exploited (‘Rdst’), and drawdown of resources one and two (‘mnR1’ and ‘mnR2’). Simulation scenarios are identified using a numbering scheme where the first two digits identify the level of complementarity (‘99’ lowest level, ‘01’ highest level) and the second two digits identify the level of noise in the resource supply concentrations (‘00’ means no noise, ‘20’ means 0 to 20% noise, and ‘80’ means 0 to 80% noise).

For scenarios where the mode of reversal in the resource supply concentrations was gradual, and with no noise or 0–20% noise in the resource supply concentrations, the relationship with complementarity level was linear with a negative slope (Fig. 4a). That is, lower time-averaged richness occurred at higher levels of complementarity. When noise in the resource supply concentrations was 0–80%, the relationship became curvilinear (or weakly unimodal) where the high time-averaged richness values previously observed at low complementarity levels did not occur. This trend weighted heavily on principal component 1 (~48% of total variability) in the PCA analysis focused on gradual reversal scenarios (Fig. 6).

For time-averaged evenness, the highest and lowest values of ~0.85 and ~0.50 occurred when the mode of reversal in the resource supply concentrations was sudden (Fig. 4b). When the mode of reversal was gradual, the highest time-averaged evenness observed was ~0.75 and the lowest observed was ~0.55. Only addition of noise to the resource supply concentrations at the 0–80% amount had a pronounced effect, and this was only observed when the mode of reversal in the resource supply concentrations was sudden and complementarity level low to intermediate. Under these scenarios, an ~73% increase in time-averaged evenness occurred compared to similar sudden reversal scenarios with no noise and 0–20% noise in the resource supply concentrations.

The shape of the curve describing the relationships between complementarity level and time-averaged evenness varied depending on the mode of reversal in the resource supply concentrations (Fig. 4b). When resource supply reversals were sudden, the relationship was concaved. The higher evenness values at higher levels of complementarity in these simulations weighted heavily on principal component 1 (Fig. 5, ~57% of total variability). When resource supply reversals were gradual, the relationship was unimodel. This resulted in ~45% greater time-averaged evenness when the mode of reversals were gradual and the level of complementarity intermediate.

**Fig 7:**
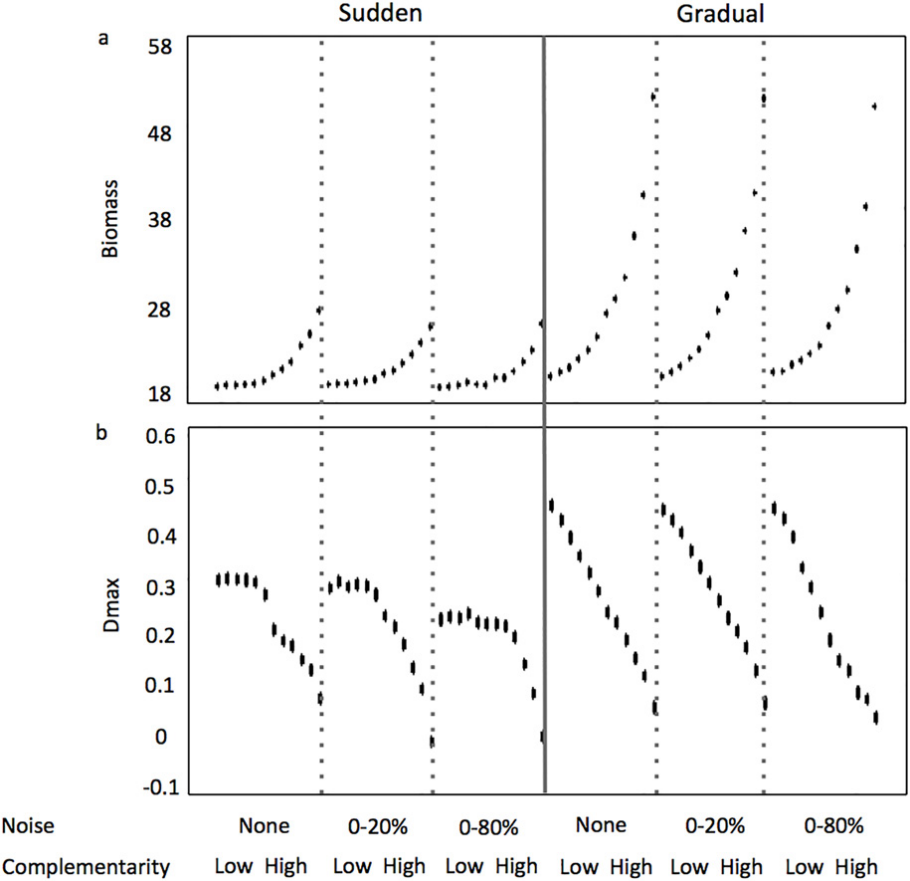
Variability of Biomass (a sum of co-existing populations’ cell density, in units of x10^6^ cells liter^-1^) (a) and transgressive overyielding index Dmax (b) along a gradient of increasing complementarity in the assemblages and noise in resource concentration from the supply, when the reversal in resource supply concentrations is sudden and gradual. Error bars represent 95% confidence intervals.

Concerning time-averaged biomass, the mode of reversal in the resource supply concentration again explained the most variance. But different from above, the mode of reversal was followed by the level of complementarity, then the amount of noise in the resource supply concentrations, as variance explanatory factors (Table 1). In scenarios where the mode of reversal in the resource supply concentrations was sudden, maximum time-averaged biomass was ~28 x10^6^ cells liter^-1^. When the mode of reversal was gradual, the time-averaged biomass maximum was ~85% greater, ~52 x10^6^ cells liter^-1^ (Fig. 7a). In all scenarios, the shape of the curve describing the relationship between complementarity level and time-averaged biomass was exponentially increasing where minima of ~18–20 x10^6^ cells liter^-1^ occurred at the lowest levels of complementarity. This trend weighted heavily on the first principal component in the PCA analyses for sudden and gradual reversal scenarios (~57% and ~48% of total variability, respectively) (Figs. 5 and 6).

Different from three assemblage characteristics mentioned above, the level of complementarity explained the most variance in time-averaged overyielding. The level of complementarity was followed closely by the mode of reversal in the resource supply concentrations, then the amount of noise in the resource supply concentrations, as variance explanatory factors (Table 1). The lowest levels of complementarity resulted in the greatest values of time-averaged overyielding, where at the most they were 6- to 7-fold greater than time-averaged overyielding at the highest levels of complementarity (Fig. 7b). This trend weighted heavily on the first principal component in the PCA analyses for sudden and gradual reversal scenarios (~57% and ~48% of total variability, respectively) (Figs. 5 and 6). The mode of reversal in the resource supply concentrations influenced the shape of the curve describing the relationship between complementarity level and time-averaged overyielding, where scenarios with sudden reversals showed a curvilinear relationship with maxima ~0.25–0.32, and scenarios with gradual reversals showed a linear relationship with a negative slope and maxima ~0.45–0.47. These maxima occurring in scenarios with gradual reversals were ~75% greater than those occurring in the sudden reversal scenarios. The amount of noise in the resource supply concentrations only notably influenced time-averaged overyielding (decreased by ~28%) in scenarios where the mode of reversal was sudden and complementarity was low, and only when the amount of noise was 0–80%.

**Fig 8:**
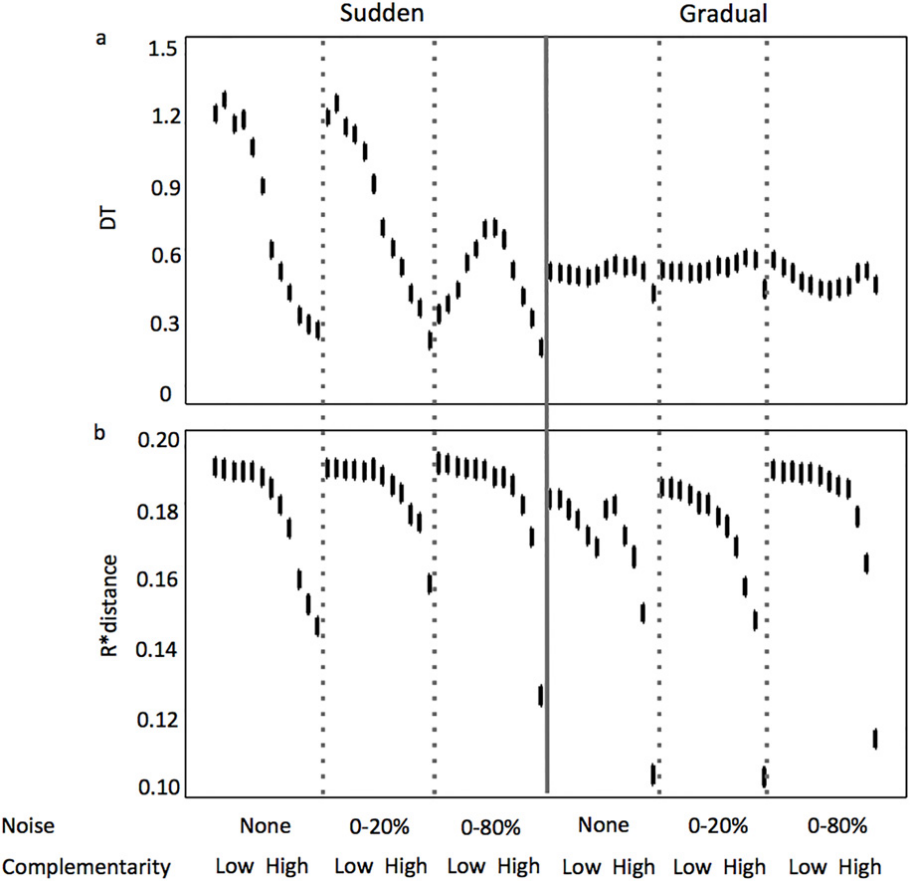
Variability of species interactions D_T_ (a) and R* Distance (b) along a gradient of increasing complementarity in the assemblages and noise in resource concentration from the supply, when the reversal in resource supply concentrations is sudden and gradual. Error bars represent 95% confidence intervals.

Like richness and evenness, the mode of reversal in the resource supply concentration explained the most variance in time-averaged species interactions, followed by the amount of noise in the resource supply concentrations, then followed by the level of complementarity (Table 1). The highest and lowest time-averaged species interaction values of ~1.3 and ~0.2 occurred when the mode of reversal in the resource supply concentrations was sudden. When the mode of reversal was gradual, values were relatively unchanging, ~0.5 (Fig. 8a). Only addition of noise to the resource supply concentrations at the 0–80% amount had a pronounced effect, and this was only observed when the mode of reversal in the resource supply concentrations was sudden and complementarity level low to intermediate. Under these scenarios, an ~66% decrease in time-averaged species interactions occurred compared to similar sudden reversal scenarios with no noise and 0–20% noise in the resource supply concentrations. This trend of higher species interactions at lower levels of complementarity when there was no noise or 0–20% noise in the resource supply concentrations weighted heavily on principal component 1 (~57% of total variability) in the PCA analysis focused on sudden reversal scenarios (Fig. 5). When the mode of reversal in resource supply concentrations was sudden, the relationship between complementarity level and time-averaged species interactions was either linear with a decreasing slope (no noise and 0–20% noise amounts) or unimodel (0–80% noise amount). When the mode of reversal in resource supply concentrations was gradual, time-averaged species interactions was insensitive to complementarity level.

Like time-averaged overyielding, the level of complementarity explained the most variance in time-averaged R*distance. The level of complementarity was followed closely by the mode of reversal in the resource supply concentrations, then the amount of noise in the resource supply concentrations, as variance explanatory factors (Table 1). The lowest levels of complementarity resulted in the greatest values of time-averaged R*distance (Fig. 8b), where at the most they were 19-fold greater than time-averaged R*distance at the highest levels of complementarity (Fig. 8b). This trend weighted heavily on the second principal component in the PCA analyses for sudden reversal scenarios (~28% of total variability, Fig. 5), and on the first principal component in the PCA analyses for gradual reversal scenarios (~48% of total variability, Fig. 6). The shape of the curve describing the decreasing relationship between complementarity level and time-averaged R*distance was similar between the two mode of reversal scenarios, and the amount of noise in the resource supply had little effect.

**Fig 9:**
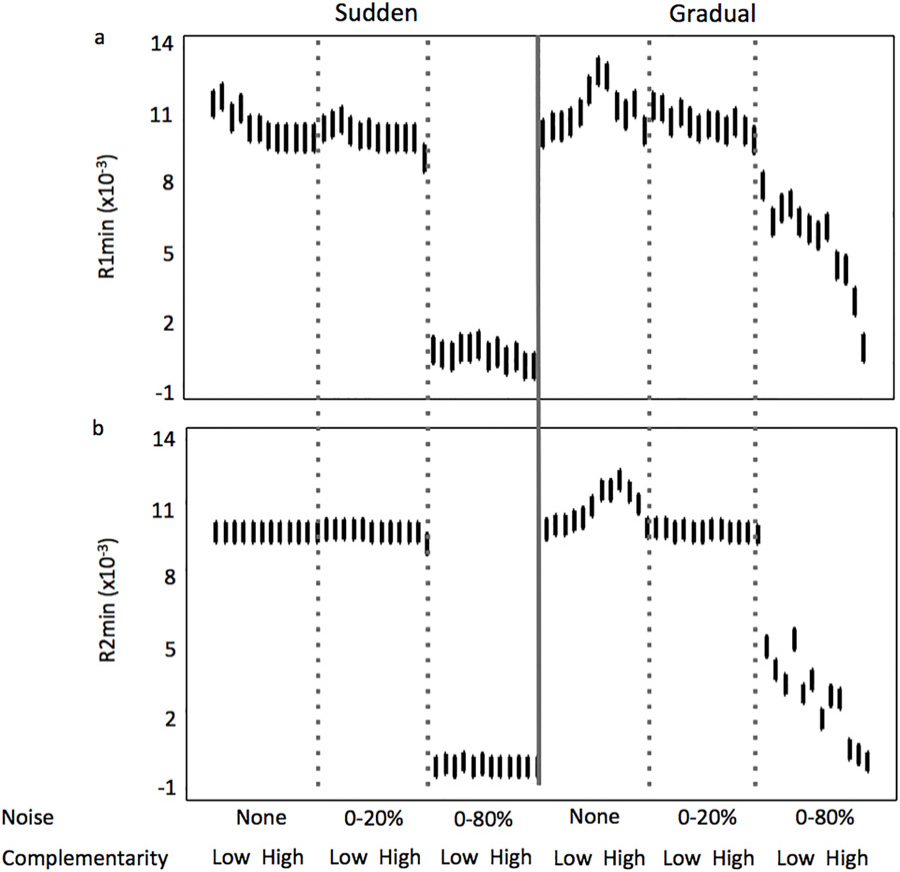
Variability of minimum in Recourse 1 (a) and Resource 2 (b) along a gradient of increasing complementarity in the assemblages and noise in resource concentration from the supply, when the reversal in resource supply concentrations is sudden and gradual. Error bars represent 95% confidence intervals.

The amount of noise in the resource supply explained the most variance in time-averaged resource exploitation (R1min and R2min), followed by the mode of resource reversal, then by the level of complementarily (Table 1). Resource exploitation was greatest when noise in the resource supply was highest (Fig. 9a, b), a relationship showed strongly on the second principal components for both sudden and gradual resource supply reversal scenarios (Figs. 5 and 6). When considering each of the noise levels independently, resource drawdown was more consistent and lower in the sudden resource reversal scenarios compared to the gradual resource reversal scenarios. A trend between resource drawdown and complementarity level was only observed at the highest noise level in simulations where resource reversals were gradual.

**Fig 10:**
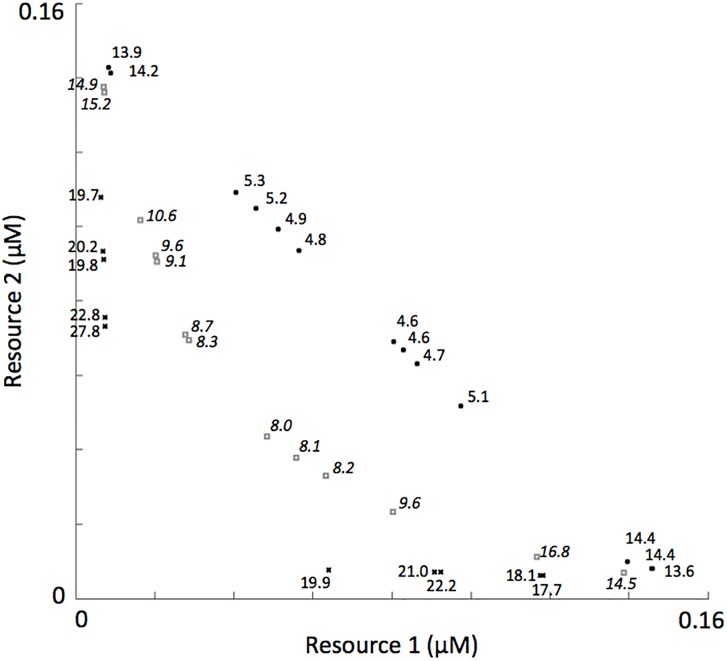
Representative R* species distributions from self-organized assemblages where the initial species pools had the lowest (filled circles), intermediate (open squares) and highest (crosses) levels of complementarity. The numbers associated with each marker is the time-averaged biomass of that species when in monoculture.

Finally, the level of complementarity influenced the distribution of species along the resource trade-off curve (Fig. 10). For example, a more even distribution of species across the resource trade-off curve tended to emerge during self-organization at low levels of complementarity, while species tended to cluster at either extreme of the resource trade-off curve when complementarity was high. Lastly, species with R*s positioned closer to the origin produced higher yields when in monoculture under these recurrently fluctuating resource supplies.

## Discussion

High species richness resulted in almost all of our simulations, which is consistent with theory regarding fluctuating environments [11, 12] and many previous studies focused on phytoplankton [50–52]. Exceptions only occurred when transitions in the resource supply were sudden, noise level in resource supply concentration was high, and complementarity was low. In those exceptional simulations, two species emerged from the self-organization process. Because high richness was the norm in our simulations, it is likely that in coastal systems characteristic of multiple limiting resources [5–10] the process of recurrent resource fluctuations contributes to species richness.

Richness was higher in our simulations when transitions in resource supply concentrations were gradual compared to sudden. Intuitively, slower change in resource supply concentrations would lead to a slower progression of the system condition along the resource trade-off curve. In other words, available resources would occur at ratios other than the conditions at the extremes of the resource trade-off curve for greater periods. This likely enabled species characteristic of intermediate R*s to perform better in simulations where transitions in resource supply concentrations were gradual.

Interestingly, richness was lower when complementarity among the members of an assemblage was high. The reasons for this were two-fold. First, species clusters emerged at either extreme of the resource trade-off curve when complementarity was high. As shown previously [33, 53], and also in our model, species occurring within clusters have similar competitive abilities for at least one of the resources. Species unassociated with a cluster experience intense competition with species associated with clusters, and are frequently excluded [53]. That is also what happened in our simulations during the period of self-organization when complementarity was high. Our model adds to this understanding by showing that the emergence of species clusters leads to an overall decrease in competitive interactions after the process of self-organization is completed, especially when transitions in resource supply concentrations are sudden. The second reason for lower richness at high complementarity is a compression of the niche. The distance between R*s of species at the extremes of the resource trade-off curve decreased through the process of self-organization, thereby lessening the chance for niche-partitioning.

In simulations where transitions in resource supply were sudden, lower richness occurred with low complementarity. Lower richness also occurred with low complementarity in simulations where noise was applied to the resource supply concentration. This relationship can be explained by the poorer performance in monoculture of species with intermediate R*s from assemblages with lower complementarity compared to those of higher complementarity. For example, time-averaged biomass was ~5 x 10^6^ cells liter^-1^ for species of intermediate R*s when complementarity was at its lowest level, while time-averaged biomass was ~20 x 10^6^ cells liter^-1^ for species of intermediate R*s when complementarity was at its highest level. Species with intermediate R*s that are poor performers in monoculture would not likely do well in a mixed assemblage exposed to dynamic shifts in the supply of resources. This explains why richness was lower in our simulations with sudden transitions in resource supply concentrations, and in our simulations with noise applied to the resource supply concentrations.

The differing processes acting to lower richness at either extreme of the complementarity gradient explored here (emergence of species clusters and niche compression when complementarity was highest, and poor-performing species of intermediate R*s when complementarity was lowest) led to a unimodel relationship between species richness and complementarity level. The exception to this observation was when transitions in resource supply concentrations were gradual and no noise was applied to the resource supply concentrations. Our theoretical observation of a unimodel relationship between species richness and complementarity in most of our scenarios is not consistent with some previous studies reporting a positive relationship [28, 54, 55]. It may be, however, that those previous studies did not investigate this relationship in systems characteristic of species clusters or where the niche is compressed. In other words, our theoretical observations may have extended the level of complementarity beyond what those previous studies addressed.

The highest biomasses occurred when complementarity levels were highest. Interestingly, only in the scenarios were transitions in resource supply concentration were gradual and noise in the resource supply concentration high did accumulated biomass relate to resource drawdown. This demonstrates that in recurrently fluctuating environments the effect of complementarity among members of an assemblage can be realized even when resources are not fully exploited. To the best of our knowledge, this is the first reporting of this phenomenon.

In all of our simulations, transgressive overyielding resulted. Two conditions contributed to this finding. First, the recurrent environmental fluctuations did not favor accumulation of biomass in monocultures. And second, matured assemblage structures likely have a greater incidence of overyielding than immature assemblages. Regarding monoculture performance, a fluctuating ratio in the resource supply creates an environment where ambient nutrients oscillate towards and away from the monoculture’s optimal resource ratio. Accumulation of biomass would likely lessen over time if the ambient resource ratio diverged a great distance from the monoculture’s optimum. In an assemblage, however, there are many species distributed along a resource gradient. As the ambient resource ratio changes, a succession of species ensues allowing optimal use of resources [41]. Because of this, resources would be used to a greater extent and biomass accumulation of the assemblage would be greater over time. This mechanism contributed to the consistent observation of transgressive overyielding in our simulations. Regarding the state of assemblage maturation, long-term field experiments suggested complementarity effects grew stronger through time as communities developed [25–27]. It was also suggested that strong diversity effects and resource partitioning may not fully develop over short periods, thus reducing the magnitude of overyielding [56]. In our simulations, transgressive overyielding emerged as a result of self-organization over a period of 15 years, encompassing many 1000s of generations. It seems, therefore, that overyielding may be more likely in systems characterized by recurrent environmental fluctuations that have been acting in the long term, such as coastal lagoons, estuaries and bays.

Paradoxically, transgressive overyielding in the self-organized assemblage was least at the highest levels of complementarity in the initial species pool, and consequently least when biomass was highest. Recall, few to no species of intermediate R*s comprised these assemblages. The assemblage members were mostly species within clusters located nearer to the extremes of the resource trade-off curve. These species were characteristic of lower R* for either of the resources and thus performed better in monoculture relative to species with intermediate R*s (this led to higher values of *M*_*i*_ in equation 8, thereby reducing the values of *D*_*max*_). Consequently, the degree of transgressive overyielding was lessoned. These model observations, i.e., inverse relationship between overyielding and biomass, underscores the challenges of using the overyielding metric to compare assemblages self-organized from different initial species pools.

Our findings illustrate how the mode of transition between reproductive growth limiting nutrients in fluctuating environments might be important to various aspects of phytoplankton assemblages. Furthermore, our findings illustrate how the effects of human population growth and associated watershed development (i.e., construction of dams and increased interbasin water transfers) might be complex. For example, with human population growth and development a shift from sudden towards gradual transitions in resource supplies is anticipated [23, 24]. As mentioned previously, this would occur because during reservoir operations peak in-stream flows tend to be lower relative to historical (pre-dam) conditions and low in-stream flows tend to be elevated, resulting in a less dynamic flow regime [23, 24]. Similarly, during interbasin water transfers flow through the watershed receiving the water can be elevated, while flow through the watershed donating the water can be reduced, with both scenarios resulting in a less dynamic condition.

The possible effects of shifts towards gradual resource supply fluctuations from sudden fluctuations on phytoplankton assemblages will be dependent, in part, on the level of complementarity among species comprising assemblages. For example, if complementarity is low, then an increase in species richness with a possible decrease in species interactions might ensue, with biomass remaining nearly the same and an increase in the level of overyielding. If complementarity is high, then slight increases in richness along with slight decreases in species interactions might follow, but now with large increases in biomass and similar levels of overyielding. If complementarity is intermediate [42], then similar richness (sometimes slight increases) along with decreases in species interactions might result, along with an increase in biomass and similar levels of overyielding.

While these findings provide insights into how the mode of fluctuation in resource supply influences phytoplankton, there is still much to be learned by building into this model framework the influences of temperature, stratification, light, grazing and other processes important to phytoplankton assemblage structure. It would also be beneficial to include variable maximum reproductive growth rates and more dynamic inflows. There will certainly be interaction effects, as many of these processes are not independent from each other, which underscores the importance of applying numerical models to aid in unraveling this complexity. From the work presented here, it seems very likely that an understanding of the processes that influence the level of complementarity in phytoplankton assemblages will be paramount, enabling a better understanding of how human population growth and watershed development will impact environments of the coastal zone, such as lagoons, estuaries and bays. This information will also likely be useful for landscape-scale management approaches aimed at promoting healthy coastal ecosystems, as the timing and magnitude of in-stream flows in watersheds replete with reservoirs can be regulated, to a degree.

## Supporting Information

S1 Assemblage Data Shown in Figs. 4, 7, 8 and 9 where the level of complementarity was determined: a) with a = 0.99, b) with a = 0.82, c) with a = 0.66, d) with a = 0.50, e) with a = 0.39, f) with a = 0.28, g) with a = 0.17, h) with a = 0.13, i) with a = 0.09, j) with a = 0.05, k) with a = 0.03, and l) with a = 0.01. (XLSX)
